# Effects of using a snooze alarm on sleep inertia after morning awakening

**DOI:** 10.1186/s40101-022-00317-w

**Published:** 2022-12-31

**Authors:** Keiko Ogawa, Emi Kaizuma-Ueyama, Mitsuo Hayashi

**Affiliations:** 1 Integrated Arts and Human Sciences Program, Graduate School of Humanities and Social Sciences, Hiroshima University, Higashi-Hiroshima, Hiroshima, Japan; 2 School of Integrated Arts and Sciences, Hiroshima University, Higashi-Hiroshima, Hiroshima, Japan

**Keywords:** Sleep loss, Snooze alarm, Awakening, Sleep inertia, Normal nocturnal sleep, Polysomnograms

## Abstract

**Background:**

Many people use the snooze function of digital alarm clocks for morning awakening, but the effects of a snooze alarm on waking are unclear. We examined the effects of a snooze alarm on sleep inertia, which is a transitional state characterized by reduced arousal and impaired cognitive and behavioral performance immediately upon awakening.

**Methods:**

In study 1, healthy Japanese university students responded to a sleep survey during a psychology class (study 1), and we collected 293 valid responses. In study 2, we compared a separate sample of university students (*n* = 10) for the effects of using or not using a snooze alarm on sleep inertia immediately after awakening from normal nocturnal sleep in a sleep laboratory.

**Results:**

Of 293 valid respondents in study 1, 251 often used a tool to wake up in the morning (85.7%). Moreover, 70.5% reported often using the snooze function of their mobile phones, mainly to reduce anxiety about oversleeping. Study 2 indicated no differences in the sleep quality or quantity before awakening with or without the snooze alarm, except in the last 20 min. However, during the last 20 min of sleep with snooze alarm, the snooze alarm prolonged waking and stage N1 sleep. Stage N1 sleep is non-rapid eye movement sleep that is primarily defined as a drowsy state. Furthermore, Global Vigor values were enhanced after awakening compared to pre-sleep in the no-snooze condition.

**Conclusions:**

Using a snooze alarm prolongs sleep inertia compared to a single alarm, possibly because snooze alarms induce repeated forced awakenings.

## Introduction

Today, the nocturnal sleep duration has become shorter for most people. The Japan Broadcasting Corporation (NHK) reported in 2020 that the mean sleep time of Japanese people was 7 h 12 min [1], down from 8 h 13 min in 1960 [2]. Sleep loss (deprivation) has led to increased daytime sleepiness and bad moods on awakening [3], with impairments of specific cognitive function [4, 5] and decreased immune, inflammatory and cardiovascular functions [6]. Moreover, sleep loss (deprivation) increases sleep inertia [7, 8], which is a transitional state of reduced arousal and impaired cognitive and behavioral performance [7–11] immediately upon awakening.

Sleep inertia appears after normal nocturnal sleep [8, 11], and its duration rarely exceeds 30 min [8]. Many factors increase the intensity and duration of sleep inertia. These factors include prior sleep deprivation [8], the length of waking time prior to sleep [12], the sleep stage at awakening [7, 13], circadian timing of awakening [14, 15], the amount of non-rapid eye movement (NREM) slow-wave sleep (SWS) [10], and the method of waking [11, 16, 17]. Sleep deprivation studies on the relationship between sleep loss and sleep inertia have indicated that sleep inertia worsens on partial sleep deprivation (2 h) nights compared to sufficient sleep (8 h) nights [7]. Moreover, McHill et al. [14] reported that performance was worse after chronic restricted sleep when sleep opportunities for a 24-h day were 5.6 h compared to normal sleep when sleep opportunity for a 24-h day was 8 h. Research on the relationship between sleep stages and sleep inertia has reported that performance decreased upon waking from SWS sleep compared to N1, N2, and REM sleep [13, 18, 19]. Studies on the effect of endogenous circadian cycles on sleep inertia indicated that sleep inertia is worse after nights when the core body temperature is lower than the day [14, 15]. Studies have also suggested that the effect of sleep stages and circadian timing on sleep inertia was influenced by sleep loss (deprivation) [20].

Reducing sleep inertia helps people awaken refreshed in the morning. Research on factors influencing sleep inertia has suggested strategies for reducing sleep inertia, including sleeping for an optimal duration and waking up from light sleep. Moreover, self-awakening (SA), a method of waking up at a predetermined time without an alarm [11, 16, 17, 21], is an effective strategy for reducing sleep inertia. People who plan to SA show increasing sympathetic nervous activity before waking up, suggesting that the body prepares for waking up before sleep termination [22, 23]. For example, Kaida et al. [16] observed that the heart rate gradually increased before SA and suggested that the increased heart rate facilitates a smoother transition from sleep to awakening. Allen [24] indicated that adrenocorticotropin release before awakening, which facilitates sympathetic nervous activity, reduces sleep inertia. In Japan, only 10.3% of university students and 18.9% of workers reported SA [17]. Mattingly et al. [25] investigated the effect of sleep duration on the waking method of 385 full-time workers in the USA. They reported that the sleep duration on natural wake days (mean sleep duration: 8.74h ± 3.77 min) was significantly longer than on days when participants used an alarm or a snooze alarm to wake up (mean sleep duration: 7.83h ± 3.54 min for the alarm and 7.95 h ± 3.66 min for the snooze alarm). Studies on Japanese people have suggested that only a few Japanese use SA [17] because they have a short sleep time [26, 27]. It is also possible that many Japanese people use external tools such as alarms to wake up in the morning because of sleep loss and difficulties in awakening.

The snooze function of alarm clocks helps avoid oversleeping after turning off the alarm. Some people use the snooze function repeatedly after the first alarm before they awaken, presumably to awaken gradually and gently. Mattingly et al. [25] reported that 57% of 450 participants habitually used a snooze alarm, and they (snoozers) had a lighter sleep and a higher resting HR across the whole night and in the last hour of sleep than no-snoozers. Self-awakening and sleep inertia studies have shown that light sleep [13, 18, 19] and increased HR before awakening [24] effectively reduce sleep inertia. On the other hand, Mattingly et al. [25] expressed concern that lighter sleep across the whole night over the long term might lead to chronic sleep loss. Moreover, an increased resting HR is associated with numerous adverse health effects, including diabetes, heart disease, and mortality. Therefore, Mattingly et al. concluded that research is needed to clarify the effect of the snooze function on human health and sleep.

The present study investigated the efficacy of the snooze function in alarm clocks on sleep inertia. We conducted a two-part study in which study 1 surveyed participants on the prevalence of using snooze alarms and the daily settings of snooze alarms. Then, study 2 examined the effects of using a snooze alarm on sleep inertia after awakening from nocturnal sleep in a smaller group of participants in a sleep laboratory. We hypothesized that sleep inertia would decrease in the snooze condition more than in the no-snooze condition if the physiological effects of snooze alarms were similar to self-awakening.

## Method: study 1

### Participants

Japanese university students (*n* = 296, 169 women; 127 men: age range 18–28 years) attending a psychology class responded to a questionnaire inquiring about their sleeping and waking habits. The research protocol of this study was approved by the Research Ethics Committee of Hiroshima University (No. 30-02). The participants were briefed about the study content, and they gave their informed consent before participating in the study.

### Procedure

Participants responded to a range of questions regarding their sleep habits and alarm clocks and mobile phone’s snooze functions use on a four-point scale comprising 1 (often), 2 (sometimes), 3 (few times), or 4 (never). The questions included “How many minutes before the actual waking time do you set the alarm?” “How many times do you hit the snooze button between the first and the last alarm (the wake-up time)?” The participants also gave free responses to the question, “Why do you use the snooze function?”

## Results: study 1

Tables 1 and 2 show the number of participants using a tool to wake up in the morning. Data from three participants were removed from the analysis due to missing values. Of the remaining 293 participants, 251 participants (85.7%) reported they often used a tool to wake up in the morning, and fewer students reported that they used a tool “sometimes” (9.6%), “a few times” (3.4%), or “never” (1.4%; *n* = 4) (Table 1). Moreover, 204 (70.5%) among 289 students, after excluding those who never used a tool to wake up, reported that they often used the phone’s snooze function, and fewer students reported that they did so sometimes (9.0%), a few times (6.3%) or never (14.2%; *n* = 41) (Table 1). Among the snooze function users (*n* = 248), 47.5% reported using it seven times a week, 10.0% six times, 26.7% five times, and 15.9% less than four times (Table 2). The two main reasons for using the snooze function were concerns about not awakening on time with a once-only alarm (50.7%) and the desire to feel secure about waking up before going to sleep (35.6%).

**Table 1:** Participants’ using a tool and the snooze function to wake up

	External tool (*N*= 293)	Snooze (*N* = 289)
Often	251	204
Sometimes	28	26
A few times	10	18
Never	4	41

**Table 2:** Participants’ use of different snooze settings

Use per week		Snooze period	
(*N* = 248)		(*N* = 211)	
7-times	118	30 min	184
6-times	25	30–60 min	24
5-times	66	60 min	3
Less than 4-times	39		

*Note*: “Snooze period” question was “How many minutes before the actual waking time do you set the alarm?”

Of the participants that reported using the snooze function, 211 responded to all the questions about the snooze settings. Among them, 184 participants (87.2%) set the snooze alarm to ring 30 min before the predetermined waking time, 11.4% set it to ring 30–60 min before the waking time, and 1.4% set it to ring over 60 min before the waking time (Table 2). The mean time for setting the snooze alarm before the predetermined waking time was 20.3 min (SE = 1.3). The mean frequency of using the snooze alarm was 6.2 (SE = 0.2) times per morning (the mode = 4 and median = 5). The mean interval between snooze alarms was 7.0 (SE = 0.4) min (mode and median were 5 min).

## Method: study 2

Study 2 compared the effects of using or not using a snooze alarm on sleep inertia immediately after awakening from normal nocturnal sleep. The participants in study 2 did not participate in study 1.

### Participants

Study 2 used the same questionnaire as in study 1 to pre-screen ten healthy Japanese university students (5 women, 5 men: age range 21–26 years) who reported using an external tool to wake up and used a mobile phone’s snooze function 5 to 7 times per week (*M =* 6.2, SE = 0.3). We conducted a post hoc power analysis using G*Power 3.1.9.6 [28]. We assumed the standard criteria for significance (α = .05) and small-to-medium effect size (*f* = .23) for a repeated-measures analysis of variance (ANOVA). The power analysis indicated that the 1–β error probability = .47. Study 2’s protocol was approved by the Research Ethics Committee of Hiroshima University (No. 30-03). All participants gave their written informed consent to participate in the study after a briefing about its content.

### Measures

#### Global vigor (GV) and global affect (GA) scores

We assessed the participants’ Global Vigor Scores (GV) to evaluate their alertness, sleepiness, motivation, loss of effort, and weariness. We also evaluated their Global Affect Scores (GA) for happiness, sadness, calmness, and tension [29]. The participants rated their vigor and affect on the GV and GA before bedtime and after awakening using a Visual Analog Scale ranging from 0 (not at all) to 100 (absolutely) [30]. We also used the Japanese version [31] of Spielberger’s [32] State Anxiety Scale (STAI) to assess the participants’ anxiety at bedtime. Participants also completed the standardized and revised version of the Oguri-Shirakawa-Azumi (OSA) Sleep Inventory for Middle-Aged Respondents (OSA-MA, [33]) immediately after waking up. They rated the condition of five variables on the OSA-MA using a four-point semantic differential (SD) scale. The variables assessed were sleepiness on rising (items included, “I can concentrate,” “I feel a freedom,” “I have a clear head,” and “I can answer quickly.”); initiation and maintenance (items included “I slept well,” “I had a light sleep,” “I dozed a lot,” “I fell asleep quickly,” and “I woke up often.”); frequent dreaming (items included, “I had many nightmares,” and “I dreamed often.”), refreshment (items included, “I am still tired,” “I am tired all over,” “I have a good appetite,” and “I feel bad.”); sleep length (items included, “I had a long sleep.”).

#### Objective performance task

We used this task to measure the participants’ alertness objectively. The task is a simple auditory reaction time task using only one auditory stimulus (a computer-generated 66 dB beep tone). Previously studies have used this task to examine sleep inertia and vigilance [13]. The task requires a participant to press a button as quickly as possible after hearing an auditory stimulus at random intervals of 2–8 s (mean interval 5 s). One trial of this task comprised one auditory stimulus, and 60 trials comprised a task block that lasted 5 min. Participants performed one task block in the pre-sleep session and three in the post-sleep session.

#### Polysomnogram

We recorded standard polysomnograms to evaluate the participants’ sleep quality when using and not using a snooze alarm. We recorded electroencephalograms (EEGs) at four scalp sites (C3, C4, O1, O2) according to the international 10% system [34] with a time constant of 0.3 s. We recorded horizontal electrooculograms (EOG) from the outer canthi of both eyes with a time constant of 2 s. The electromyogram (EMG) was recorded from the mentalis muscles with a time constant of 0.003 seconds. We conducted the entire recording was conducted using Ag/AgCl electrodes. We used high-cut filters of 60 Hz for EEG and EOG and 120 Hz for EMG at a sampling rate of 500 Hz. Electrode impedance was below 10 KΩ. We scored sleep stages in 20-s epochs and calculated sleep variables using EEG, EOG, and EMG polysomnogram data [35–37]. We classified nocturnal sleep into four-sleep stages: stage N1 sleep (shallow non-rapid eye movement (NREM) sleep, defined as the drowsy state), stage N2 sleep (shallow NREM sleep), stage N3 sleep (deep NREM sleep), and stage REM sleep (rapid eye movement (REM) sleep).

### Procedure

Participants slept in their homes during the control night’s week. We monitored their sleep-wake schedules at home using sleep logs and wrist actigraphy (Actiwatch AW64, Mini-Mitter Co., Bed, Ore., USA) to confirm that their sleep-wake habits were constant (*M* total sleep time = 373, SE = 21.5 min; *M* bedtime = 1:29:34, SE = 0:19:22; *M* awakening time = 7:42:13, SE = 0:13:52). We used the identical sleep-wake schedules as the control nights on the experimental nights in the sleep laboratory. We also asked the participants to wake up daily during the control nights by using the snooze function of an identical mobile phone (Softbank Corp., 831P) to the one to be used later during experimental nights in the sleep laboratory to familiarize themselves with the experimental procedure. Based on the results of study 1, we set the snooze alarm to activate four times at 5-min intervals during the 20-min before a predetermined waking time. The alarm was either stopped by the participants or continued for 60 s.

After the week of control nights, the participants spent three consecutive experimental nights in a sleep laboratory. On the experimental days, we requested the participants to abstain from ingesting substances that affected sleep and wakefulness, including alcohol, caffeine, and nicotine. The first experimental night was an adaptation session, and the second and third nights were data collection nights under the snooze alarm or no-snooze alarm sleep conditions, which we counterbalanced across the participants. We fixed the awakening and going to sleep times at the participant’s usual waking and bedtimes by referring to the control nights (*M* total sleep time = 402.0, SE = 8.0 min*; M* bedtime = 1:10:00, SE *=* 0:15:55; *M* awakening time = 7:52:00 am, SE = 0:13:34).

The participants finished dinner in their homes and arrived at the sleep laboratory approximately 2 h before bedtime. Then, we attached the polysomnogram electrodes. In the laboratory, the participants could drink water freely, and we asked them to go to the toilet before bedtime. Then, the participants entered a soundproof, air-conditioned isolation unit 20 min before their predetermined sleep time. We eliminated all time cues, including natural light, from this unit. Then, the participants completed the GV and GA Questionnaires [29] and performed the simple auditory reaction time task for 5 min, 15 min before sleep. Following this, the participants responded to the STAI [31]. We instructed them to go to sleep at the usual time. Prior to the awakening time in the snooze alarm condition, a mobile phone’s snooze alarm (50 dB) kept beside the pillow was activated for 1 min, and this alarm recurred at 5-min intervals for 20 min. Participants could stop the alarm before 1 min had elapsed by pressing a button on the mobile phone. We asked the participants to sleep again until the alarm finally woke them up. In the no-snooze condition, the snooze alarm did not activate before waking up. In both conditions, participants were awakened at the predetermined awakening time by an auditory stimulus (a computer-generated 60 dB of beep tone). After waking up, the participants completed the OSA-MA sleep inventory questionnaire [33]. Then, they conducted the post-sleep sessions during 30 min after awakening: (1) the 5-min auditory reaction time task (session 1, session 2, session 3); and (2) responding to the GV and GA questionnaire using the visual analog scale for every 10 min.

### Statistical analysis

We used SPSS ver. 28 (IBM) for statistical analyses. We used *t*-tests to compare anxiety levels (STAI scores) at bedtime, sleep variables (time in bed, the total sleep time, sleep efficiency, waking time after sleep onset, sleep stage times (stages N1, N2, N3, and REM), movement time), and OSA-MA scores on snooze alarm and no snooze alarm nights. We conducted a two-factor, repeated measures analysis of variance (ANOVA) with conditions (snooze alarm and no-snooze alarm) crossed with the experimental sessions to examine task performance. We used the pre-session performance measurement before sleeping and the performance measurements of the three post-session after awakening, including auditory reaction times, correct response rates, and subjective GV and GA ratings. The ANOVA effect sizes were shown with partial η^2^ (ηp^2^). We adjusted the degrees of freedom using Greenhouse-Geisser’s epsilon if the assumption of sphericity (Mauchly’s sphericity test) was significant. The probability of significance was adjusted using Bonferroni corrections if the main effect was significant. We set the statistical significance level at 0.05 for all the analyses.

## Results: study 2

### Sleep variables

The polysomnogram data indicated that the total sleeping time of the whole night was significantly shorter in the snooze alarm condition (*M =* 387.8, SE = 7.8 min) than in the no-snooze alarm condition (*M* = 396.5, SE = 7.8 min; *t* (9) = 2.780, *p* = 0.021). Table 3 shows the sleep data, excluding the last 20 min before awakening (upper part) and the sleep data of the last 20 min (lower part) on snooze and no-snooze alarm nights. During the sleeping time excluding the last 20 min, participants slept for an average of 371.9 min (SE *=* 7.8) and 376.8 min (SE *=* 7.9), respectively, on snooze alarm and no-snooze alarm nights. There were no significant differences in sleep variables between these two conditions. The total sleep time during the last 20 min was 4 min shorter with the snooze alarm (*M* = 15.7, SE = 0.9 min) than without it (*M* = 19.7, SE = 0.2 min; *p* = 0.002). Moreover, sleep efficiency was significantly worse (*p* = 0.002), and wake time (*p* = 0.009), and stage N1 sleep (*p* = 0.006) were longer on snooze alarm nights than on no-snooze alarm nights.

**Table 3:** Polysomnogram data for total nights’ sleep (excluding the last 20 min before awakening) and the last 20 min of sleep

	Snooze use		No-snooze use		*t*	*p*
	*M*	SE	*M*	SE		
Excluding last 20 min						
Time in bed (min)	382.0	(8.0)	382.0	(8.0)	–	–
Total sleep time (min)	371.9	(7.8)	376.8	(7.9)	1.82	n.s.
Sleep efficiency (%)	97.4	(0.7)	98.6	(0.2)	1.85	n.s.
Wake after sleep onset (min)	2.5	(0.6)	1.6	(0.4)	1.85	n.s.
Stage N1 (min)	43.2	(9.8)	21.5	(6.3)	1.12	n.s.
Stage N2 (min)	192.5	(19.1)	208.9	(3.3)	0.98	n.s.
Stage N3 (min)	49.9	(8.0)	44.2	(6.2)	0.57	n.s.
Stage REM (min)	73.6	(8.0)	90.0	(2.2)	1.61	n.s.
Movement time (min)	12.6	(1.5)	12.2	(1.1)	0.31	n.s.
Last 20 min						
Time in bed (min)	20.0	–	20.0	–	–	–
Total sleep time (min)	15.7	(0.9)	19.7	(0.2)	4.35	< 0.01
Sleep efficiency (%)	78.7	(4.4)	98.5	(1.1)	4.62	< 0.01
Wake after sleep onset (min)	3.9	(1.3)	0.2	(0.2)	3.28	< 0.01
Stage N1 (min)	6.1	(1.0)	1.5	(0.7)	3.58	< 0.01
Stage N2 (min)	8.0	(1.7)	11.4	(2.7)	1.08	n.s.
Stage N3 (min)	–	–	–	–	–	–
Stage REM (min)	1.6	(1.0)	6.3	(2.6)	1.81	n.s.
Movement time (min)	0.4	(0.2)	0.5	(0.2)	0.45	n.s.

*Note*: Results of *t*-test for sleep variables on snooze and non-snooze nights

*SE* standard error

Six participants woke up in stage N2 sleep, and others had already woken up before the predetermined waking time in the snooze alarm condition. In contrast, all the participants except one remained asleep until waking time in the no-snooze alarm condition. Moreover, participants were aroused in different sleep stages in the no-snooze alarm condition, such that two participants were aroused in stage N1 sleep, five were aroused in state N2 sleep, and two were aroused in stage REM sleep, while no participants were aroused during stage N3 sleep.

The participants were more frequently aroused during the last 20 min of sleep in the snooze alarm condition (*M* = 4.1, SE = 0.77 times) than in the no-snooze alarm condition (*M* = 0.3, SE = 0.21 times; *t* (9) = 6.042, *p <* 0.001). Also, the number of sleep-stage transitions increased in the snooze alarm condition (*M* = 12.2, SE = 2.0 times) relative to the no-snooze alarm condition (*M =* 3.5, SE = 0.92 times; *t* (9) = 3.538, *p* = 0.006). The sleep stages during the 20 min before awakening indicated that stage N2 and REM sleep were stable in the no-snooze alarm condition, whereas waking and stage N1 sleep increased periodically in the snooze alarm condition.

### Objective performance

**Fig. 1:**
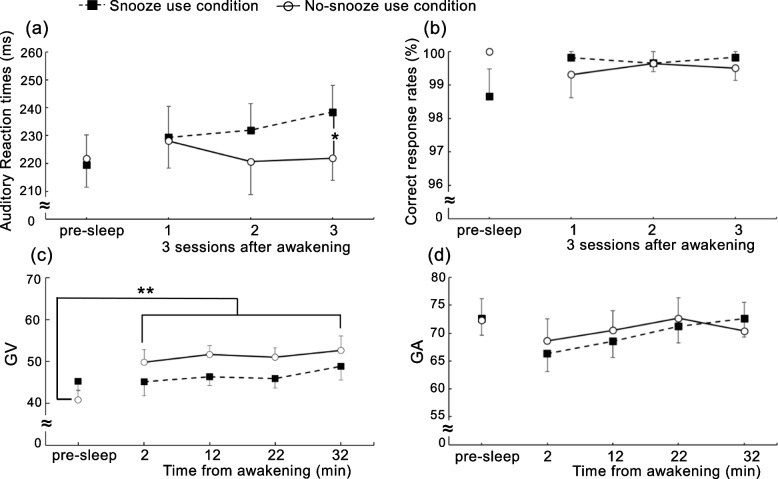
**a** Mean auditory reaction times, **b** mean correct response rates in a simple auditory reaction time task, **c** mean global vigor (GV) VAS values, **d** mean global affect (GA) VAS values before and after nocturnal sleep. *p < 0.05, **p < 0.01; snooze alarm condition vs no-snooze alarm condition. The vertical bars reflect standard errors (SE)

Figure 1a, b shows auditory reaction times (RTs) and correct response rates in the snooze and no-snooze alarm conditions. ANOVAs with the two conditions (snooze and no-snooze) crossed with the sessions (4 levels: pre-sleep, session 1, session 2, session 3) indicated neither the main effect of the condition (*F* (1, 9) = 3.39, *p* = 0.15, *η*_*p*_^*2*^ = 0.22) nor session (*F* (3, 27) = 0.87, *p* = 0.47, *η*_*p*_^*2*^ = 0.09; *W* (Mauchly’s sphericity test) = 0.327, *p* = 0.13) on auditory reaction times, whereas the interaction between condition and session was marginally significant (*F* (3, 27) = 2.57, *p* = 0.08, *η*_*p*_^*2*^ = 0.22; *W* = 0.611, *p* = 0.58). RTs were significantly longer in session 3 (*M* = 238.4, SE = 9.6 ms) in the snooze than in the no-snooze condition (*M* = 221.9, SE = 7.9 ms; *p* = 0.024). Moreover, there was no significant main effect of the correct response rates on condition (*F* (1, 9) = 0.13, *p* = 0.73, *η*_*p*_^*2*^ = 0.01) or session (*F* (1.98, 17.82) = 0.27, *p* = 0.76, *η*_*p*_^*2*^ = 0.03; *W* = 0.029, *p* = 0.00). Furthermore, the interaction between the condition and the session was not significant (*F* (1.55, 14.00) = 2.02, *p* = 0.18, *η*_*p*_^*2*^ = 0.18; *W* = 0.115, *p* = 0.01).

### Subjective reports

We analyzed STAI scores to examine the participants’ pre-sleep anxiety levels using paired *t tests* between snooze alarm (*M* = 34.6, SE = 1.6) and no-snooze alarm (*M* = 35.9, SE = 2.2) conditions (*t* (9) = 1.314, *p* = 0.221). The results revealed no significant differences in pre-sleep anxiety. Also, a series of paired *t*-tests examined post-sleep differences between snooze alarm and no-snooze alarm conditions on OSA-MA scores assessing sleepiness (*M* = 42.9, SE = 2.8 versus *M* = 43.6, SE = 2.3; *t* (9) = 0.368, *p* = 0.721); sleep-onset and sleep sustainment (*M* = 41.6, SE = 2.8 vs. *M* = 48.1, SE = 3.1; *t* (9) = 2.401, *p* = 0.040); dreaming (*M* = 46.9, SE = 3.3 vs. *M* = 44.4, SE = 3.6; *t* (9) = 0.703, *p* = 0.500); recovery from exhaustion (*M* = 42.7, SE = 2.0 vs. *M* = 44.8, SE = 2.0; *t* (9) = 2.079, *p* = 0.067); and sleep duration (*M* = 42.8, SE = 2.3 vs. *M* = 45.7, SE = 2.1; *t* (9) = 2.228, *p* = 0.053). These *t* tests indicated significant differences only for sleep-onset and sleep sustainment, with participants in the no-snooze alarm condition showing better sleep quality than those in the snooze alarm condition.

Figure 1c, d shows mean GV and GA values between snooze and no-snooze alarm conditions. We conducted repeated measures ANOVAs with two conditions (snooze and no-snooze alarm) crossed with the sessions (5 levels: pre-sleep and 2, 12, 22, 32 min after from awakening) to examine differences in GV and GA values between the conditions. The results indicated that the main effect of condition (*F* (1, 9) = 4.05, *p* = 0.08, *η*_*p*_^*2*^ = 0.31) and session (*F* (2.23, 20.08) = 1.80, *p* = 0.19, *η*_*p*_^*2*^ = 0.17; *W* = 0.047, *p* = 0.01) were not significant for GV. However, the condition by session interaction was significant (*F* (4, 36) = 4.35, *p* = 0.006, *η*_*p*_^*2*^ = 0.33; *W* = 0.170, *p* = 0.17), showing that GV was significantly enhanced after awakening (2 min: *M* = 49.9, SE = 3.0 (*p* = 0.002), 12 min: *M* = 51.7, SE = 2.1 (*p* < 0.001), 22 min: *M* = 51.1, SE = 2.3 (*p* < 0.001), 32 min: *M* = 52.6, SE = 3.5 (*p* < 0.001)) compared to pre-sleep (*M* = 40.8, SE = 2.2) in the no-snooze condition, which was not the case in the snooze condition. There was no significant difference among post four sessions. In contrast, neither the main effect of condition (*F* (1, 9) = 0.193, *p* = 0.67, *η*_*p*_^*2*^ = 0.19) nor session (*F* (2.17, 21.70) = 4.17, *p* = 0.27, *η*_*p*_^*2*^ = 0.29; *W* = 0.057, *p* = 0.01), nor the interaction between condition and session was significant for GA (*F* (2.00, 19.79) = 0.51, *p* = 0.61, *η*_*p*_^*2*^ = 0.05; *W* = 0.088, *p* = 0.02).

### Final sleep stage and objective/subjective data

Table 4 shows the number of participants that woke up from stages N1, N2, and REM sleep and those that were awake, and the number of sleep-stage transitions during the last 20 min of sleep. The number of participants who woke up from stage N2 sleep was relatively high in both snooze and no-snooze conditions. Moreover, the sleep-stage transitions were small for all sleep stages in the no-snooze than the snooze condition.

**Table 4:** Final sleep stage and objective/subjective data

	Snooze use				No-snooze use			
	N1	N2	REM	wake	N1	N2	REM	Wake
Participants (N)	0	6	0	4	2	5	2	1
Stage transition (N)	–	9.5	–	16.3	6.0	2.6	2.0	6.0
Reaction times (ms)	–	17.1	–	21.6	1.8	6.2	− 12.0	− 9.7
GV (score)	–	− 1.3	–	5.3	21.4	5.6	6.7	21.0

*Note*: Auditory reaction times were calculated by subtracting the pre-sleep session reaction times from session 3. We calculated Global Vigor scores by subtracting the pre-sleep session scores from the mean scores for all post-sleep sessions

*GV* Global Vigor

Table 4 also shows objective (auditory reaction time) and subjective (Global Vigor score) data after awakening on the sleep stages just before waking up. The auditory reaction times significantly differed between the conditions in session 3. Moreover, the GV score showed a significant difference between pre-sleep and all post-sleep sessions in the no-snooze condition. Therefore, we calculated the auditory reaction times by subtracting the pre-sleep session reaction times from session 3 and GV by subtracting the pre-sleep session scores from the mean scores in all post-sleep sessions. The results indicated that subjective reports (Global Vigor) increased after awakening from stage N1 sleep and being awake in the no-snooze condition. Moreover, the reaction times were better for all sleep stages in the no-snooze condition than the snooze condition.

## General discussion

This study investigated the efficacy of alarm clocks’ snooze function on sleep inertia. The results of study 1 indicated that 85.7% of university students in the survey used an external tool to wake up in the morning. Moreover, 70.5% of these students often used the snooze function of their mobile phones, mainly to reduce their anxiety about oversleeping. The most common snooze alarm setting reported by the students allowed 4 or 5 resets in the last 20 min of nocturnal sleep, separated by 5-min intervals. Study 2 indicated that sleep variables were not significantly different between the conditions other than in the last 20 min before the alarm sounded. However, the wake time and stage N1 sleep were prolonged after the first snooze alarm in the last 20 min of nocturnal sleep. In addition, auditory reaction times to the simple auditory task were slower in session 3, and global vigor deteriorated after awakening in the snooze alarm compared to the no-snooze alarm condition. These results suggest that the mobile phones’ snooze function might increase sleep inertia after waking up. This sleep inertia might be explained by the sleep stage before awakening and repeated forced awakening during the last 20 min of sleep.

Sleep length prior to waking [7], the circadian timing of awakening [14, 15], the time awake prior to sleep onset [12], the sleep stage at awakening [7], and the waking method [11, 16, 17] affect sleep inertia. Study 2 controlled for the time awake before sleep onset and the circadian timing of awakening under the two conditions. However, the sleep stage before awakening differed depending on the condition. Previous studies have reported that sleep inertia strongly increases after awakening from deep NREM sleep (stage N3) [38]. However, in this study, stage N3 sleep did not occur in the last 20 min of sleep (Table 3). We observed that more participants woke up from stage N2 sleep (Table 4) under both conditions. Six participants woke up from stage N2 sleep; four had already woken up before the final beeping tone in the snooze alarm condition, whereas only one had already woken up in the no-snooze alarm condition. Two participants in the no-snooze alarm condition woke up from stage N1 sleep, five from Stage N2 sleep, and two from Stage REM sleep. In the no-snooze condition, subjective reports (Global Vigor) increased in participants awakening from stage N1 sleep and participants that were awake. Moreover, the reaction times were better for all sleep stages in the no-snooze compared to the snooze condition. The effects of the sleep stages (N1, N2, and REM) before awakening on sleep inertia are especially controversial. Cavallero and Versace [13] reported that sleep inertia slowed the reaction time to a simple auditory task after waking up from stage N2 sleep compared to stage REM sleep, suggesting that sleep inertia is more pronounced after awakening from stage N2 sleep. However, Stones [39] failed to find any effects of sleep stages before awakening on sleep inertia. Koulack and Schultz [40] showed that REM sleep increased inertia. Finally, Jewett et al. [41] did not always observe the effects of sleep stages on sleep inertia. Therefore, it is difficult to make any firm conclusions about this study’s results on sleep stages.

It is also possible that the snooze alarm induces forced awakening whenever it is activated. In this study, the snooze alarm was activated four times during the last 20 min of nocturnal sleep at 5-min intervals. Participants tried to go back to sleep after each forced awakening, suggesting that the snooze alarm increased their sleep propensity and waking thresholds compared to the no-snooze alarm condition. Indeed, sleep-stage transitions increased in the snooze alarm condition (*M* = 12.2) than in the no-snooze alarm condition (*M* = 3.5). Transitions into and from sleep induce highly predictable autonomic changes [42, 43] that might discourage smooth awakening. Moreover, sleep fragmentation is related to daytime sleepiness [44] and detrimental to cogitative and vigilance tasks [45]. Therefore, sleep inertia might have increased through repeated forced awakening. According to the two-process model of sleep regulation [46] consisting of sleep homeostatic (S) and circadian rhythm (C) processes, sleepiness should be minimal when we wake up in the morning. However, there is increased sleepiness (lowered alertness and impaired performance) immediately upon awakening in the morning. Folkard and Akerstedt [47] hypothesized that sleep inertia is a third process of sleep regulation that the S or C processes of the two-process model cannot explain. Moreover, Hilditch and McHill [20] suggested that sleep inertia is an adaptive function of sleep maintenance, particularly in the morning during the last part of nocturnal sleep. Therefore, in the snooze condition, the sleep maintenance function might be enhanced by repeated forced awakening by the snooze alarm due to sleep fragmentation.

This study’s results indicated that sleep inertia only induced a significant performance difference in session 3. Sleep inertia dissipates asymptotically within 15 to 30 min after waking [8, 20]. However, we found that sleep inertia in the snooze alarm condition did not follow this dissipation curve as assessed by objective measures because behavioral performance decreased, as indicated by increased reaction times throughout the sessions. Therefore, our findings also support the hypothesis that sleep inertia is a third process that we cannot explain by the two-process sleep regulation model [47]. We might observe sleep inertia in the morning even after we have adequate sleep because sleep inertia maintains sleep in the morning, during the last part of nocturnal sleep [20, 48]. Therefore, we might always experience sleep inertia in the morning regardless of using the snooze function. For that reason, it is also possible that the snooze and no-snooze alarm conditions develop sleep inertia through similar mechanisms in the first half of sleep sessions. Moreover, sleep fragmentation in the snooze alarm condition might induce decreased sleep quality and quantity (increased waking and stage N1 sleep) during the last 20 min of sleep and continue to decrease VG (alertness, sleepiness, motivation, loss of effort, and weariness) immediately upon awakening. We suggest that prolonged sleep inertia, sleepiness, and fatigue might accumulate consistently in the snooze alarm condition, and sleep inertia might not dissipate asymptotically. As a result, we only observed a significant difference in reaction times in the last session (session 3). We suggest that future studies investigate the effect of sleep fragmentation (repeated undesired awakening) on sleep inertia (sleepiness/fatigue and behavioral performance) as a third sleep regulation process [47].

## Limitations and directions for future research

Several limitations constrain the findings of this study. Firstly, the small sample size might have limited the statistical power of the analyses. Therefore, future studies should use a larger sample. Secondly, this study found that university students use tools such as the snooze function because of their anxiety about waking up in the morning. We suggest that future studies investigate the effects of the snooze function on other age groups and professions, including full-time workers. Thirdly, Mattingly et al. [25] demonstrated that the waking method depends on the sleep duration; people with a longer sleep duration wake up naturally (self-awakening), and those with a shorter sleep duration wake up using an alarm or a snooze alarm. This study did not investigate individual waking methods based on sleep duration. Therefore, we suggest that future studies investigate this relationship. Finally, the participants knew they would not oversleep because they would be forced to wake up at the predetermined time. Therefore, the anxiety (STAI) scores before sleep were not different between this study’s experimental and control conditions. We suggest that future studies use a scenario in which the participants have to worry whether or not they can wake up in the morning. Moreover, further research is needed to examine sleep outside the laboratory, including the participants’ homes, which can provide helpful information for our daily lives.

## Conclusion

We examined the effect of using a snooze alarm to wake up in the morning on sleep inertia. Results demonstrated that 70.5% of participants often used their mobile phones’ snooze function primarily to reduce anxiety about oversleeping. However, the repeated use of the snooze alarm increases sleep inertia and fatigue after awakening. People with high anxiety and worry take longer to fall asleep [49]. In addition, sleep length and sleep latency are essential for the subjective feelings of good sleep [50]. Therefore, even though snooze alarms increase sleep inertia, they might be crucial for reducing anxiety about oversleeping and maintaining a good night’s sleep, mainly by avoiding prolonging sleep latency. Van De Werken et al. [51] showed the positive effects of artificial dawn during the last 30 min of nocturnal sleep on reducing sleep inertia. We suggest that further studies investigate appropriate strategies for using the snooze function to reduce sleep inertia and fragmentation with repeated forced awakening, including the alarm’s modality, force, volume, and interval. We must also consider countermeasures [20] for sleep inertia after awakening, including caffeine, light, sounds, music, and temperature.

## Data Availability

The datasets used and/or analyzed during the current study are available from the corresponding author on reasonable request.

## Abbreviations

ANOVA: Analysis of variance
EEG: Electroencephalogram
EMG: Electromyogram
EOG: Electrooculograms
GV: Global vigor
GA: Global affect
NHK: The Japan Broadcasting Corporation
NREM: Non-rapid eye movement
OSA-MA: Oguri-Shirakawa-Azumi Sleep Inventory for Middle-Aged Respondents
SA: Self-awakening
SE: Standard errors
SD: Semantic differential
Stage N1: Shallow non-rapid eye movement (NREM) sleep, especially defined as drowsy state
Stage N2: Shallow NREM sleep
Stage N3: Deep NREM sleep
Stage REM: Rapid eye movement (REM) sleep
STAI: State anxiety scale
REM: Rapid eye movement
RT: Reaction time
